# Open-source, fully-automated hybrid cardiac substructure segmentation: development and optimisation

**DOI:** 10.1007/s13246-023-01231-w

**Published:** 2023-02-13

**Authors:** Robert N. Finnegan, Vicky Chin, Phillip Chlap, Ali Haidar, James Otton, Jason Dowling, David I. Thwaites, Shalini K. Vinod, Geoff P. Delaney, Lois Holloway

**Affiliations:** 1 Northern Sydney Cancer Centre, Royal North Shore Hospital, St Leonards, NSW Australia; 2 Institute of Medical Physics, School of Physics, University of Sydney, Sydney, NSW Australia; 3 Ingham Institute for Applied Medical Research, Liverpool, NSW Australia; 4 Liverpool Cancer Therapy Centre, South Western Sydney Local Health District, Liverpool, NSW Australia; 5 South Western Sydney Clinical School, University of New South Wales, Sydney, NSW Australia; 6 CSIRO Health and Biosecurity, The Australian e-Health and Research Centre, Herston, QLD Australia; 7 School of Mathematical and Physical Sciences, University of Newcastle, Newcastle, NSW Australia; 8 Radiotherapy Research Group, Leeds Institute of Medical Research, St James’s Hospital and University of Leeds, Leeds, UK; 9 Centre for Medical Radiation Physics, University of Wollongong, Wollongong, NSW Australia

**Keywords:** Cardiotoxicity, Image segmentation, Breast cancer, Lung cancer, Radiotherapy, Deep learning, Cardiac substructures

## Abstract

**Abstract:**

Radiotherapy for thoracic and breast tumours is associated with a range of cardiotoxicities. Emerging evidence suggests cardiac substructure doses may be more predictive of specific outcomes, however, quantitative data necessary to develop clinical planning constraints is lacking. Retrospective analysis of patient data is required, which relies on accurate segmentation of cardiac substructures. In this study, a novel model was designed to deliver reliable, accurate, and anatomically consistent segmentation of 18 cardiac substructures on computed tomography (CT) scans. Thirty manually contoured CT scans were included. The proposed multi-stage method leverages deep learning (DL), multi-atlas mapping, and geometric modelling to automatically segment the whole heart, cardiac chambers, great vessels, heart valves, coronary arteries, and conduction nodes. Segmentation performance was evaluated using the Dice similarity coefficient (DSC), mean distance to agreement (MDA), Hausdorff distance (HD), and volume ratio. Performance was reliable, with no errors observed and acceptable variation in accuracy between cases, including in challenging cases with imaging artefacts and atypical patient anatomy. The median DSC range was 0.81–0.93 for whole heart and cardiac chambers, 0.43–0.76 for great vessels and conduction nodes, and 0.22–0.53 for heart valves. For all structures the median MDA was below 6 mm, median HD ranged 7.7–19.7 mm, and median volume ratio was close to one (0.95–1.49) for all structures except the left main coronary artery (2.07). The fully automatic algorithm takes between 9 and 23 min per case. The proposed fully-automatic method accurately delineates cardiac substructures on radiotherapy planning CT scans. Robust and anatomically consistent segmentations, particularly for smaller structures, represents a major advantage of the proposed segmentation approach. The open-source software will facilitate more precise evaluation of cardiac doses and risks from available clinical datasets.

**Graphical abstract:**

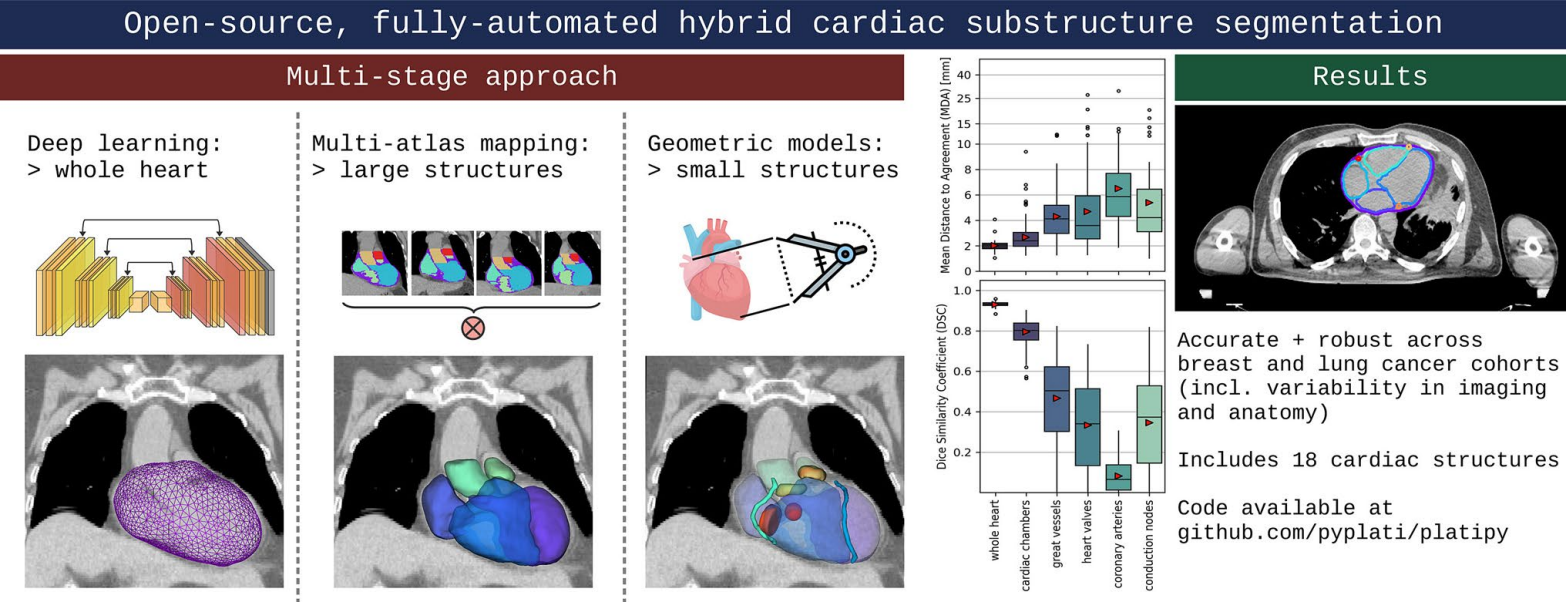

**Supplementary Information:**

The online version contains supplementary material available at 10.1007/s13246-023-01231-w.

## Introduction

Radiotherapy (RT) planning involves evaluation of risks versus benefits of planned dose to target volumes versus resulting dose to organs at risk (OARs) for toxicity. In thoracic and breast RT, automatic segmentation of cardiac substructures is necessary for the analysis of large datasets in order to develop improved cardiotoxicity risk models [1–7]. Suitability of these tools is dependent not only on delineation accuracy, but also on anatomically-consistent definitions of cardiac structures, robustness and reliability (including for smaller and low-contrast structures that are difficult to delineate manually), and software availability [8–11]. Additionally, potential to translate segmentation tools into the clinical RT workflow is important: risk models derived using specific definitions of cardiac substructures can only be applied prospectively if the same definitions are used, and since manual contouring is challenging, time consuming, and subject to intra- and inter-observer errors, automation is highly desirable.

Recent studies have demonstrated the potential of machine learning methods to automatically and accurately delineate cardiac substructures [12–20]. Existing methods can require large training datasets, fail to provide practicable segmentations for smaller structures, or rely on imaging not typically used in routine RT. Considering these factors, along with limitations in manual contouring, the aim of this work was to develop an automated approach to define a comprehensive set of 18 cardiac structures accurately, reliably, and consistent with anatomical definitions. Additionally, a focus of this work was to develop an approach that can be used with variable CT scans representing different patient cohorts, inclusive of common imaging artefacts, of varying resolution, slice thickness, use of contrast, and acquired from both 3D and 4D protocols. In this study, we detail a hybrid approach that combines deep learning (DL) segmentation, a new multi-atlas mapping algorithm extending our previous work [21–23], and a set of novel geometric modelling tools to accurately and consistently delineate cardiac substructures on highly variable CT scans, including some structures that are problematic to contour manually.

## Materials and methods

**Fig. 1:**
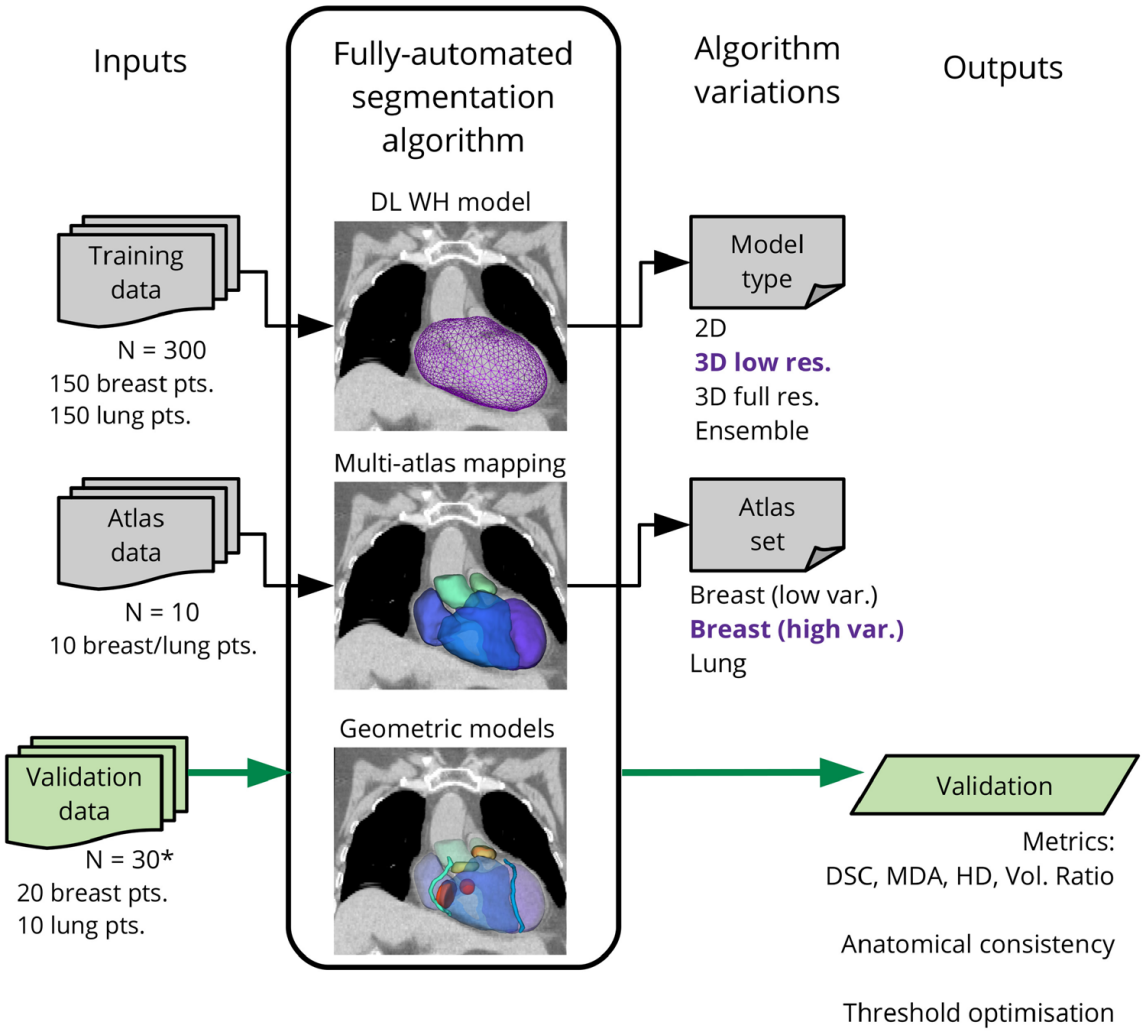
Overview of the study design. Variations to several components of the fully-automated segmentation algorithm were evaluated to find optimal configurations, and the overall process was validated using 30 cases. For definitions of acronyms please see the text. *This was performed using a leave-one-out analysis: to generate automatic segmentations for cases in the optimal atlas set this case was excluded

An overview of the study design is presented in Fig. 1. The following sections describe the data used in this study, the design and implementation of the automatic segmentation approach, and the analysis used in validation.

### Patient data

For training a DL model for whole heart (WH) segmentation (detailed in Sect. 2.2), a dataset of 300 CT scans and corresponding WH contours were extracted from a local clinical RT database, comprising data from 150 breast cancer and 150 lung cancer patients treated between 2014 and 2018. The WH volume was manually contoured by either a radiation therapist or radiation oncologist using local protocols. These protocols are consistent with published contouring guidelines [24], however there is unavoidable inter- and intra-observer variation due to differences in interpretation of these guidelines.

A separate set of 30 CT scans was used to develop and optimise the substructure segmentation model. This dataset was also obtained from the local clinical RT database, and comprised 20 non-contrast scans from breast cancer patients [21] (hereafter, the breast atlas set), on which three independent observers (medical students) contoured the heart and cardiac substructures, and an additional 10 CT scans from lung cancer patients (hereafter, the lung atlas set) on which a single observer (radiation oncologist) contoured these same volumes. All contours were further verified by a cardiologist prior to inclusion in this study. The cases from lung cancer patients were specifically selected for variations in imaging, such as image artefacts, use of contrast agent, and anatomical variations known to affect performance of automatic heart segmentation [25].

Contouring of the 30 scans was performed with reference to existing RT-specific guidelines [24]. The contoured structures included the WH, four cardiac chambers: left atrium and ventricle (LA and LV), right atrium and ventricle (RA and RV), bases of the great vessels: ascending aorta (AA), pulmonary artery (AA), superior vena cava (SVC), four coronary arteries: left anterior descending coronary artery (LAD), left circumflex artery (LCX), left main coronary artery (LMCA), right coronary artery (RCA), and heart valves: aortic valve (AV), pulmonary valve (PV), mitral valve (MV), tricuspid valve (TV). The conduction nodes (AVN and SAN) were not manually contoured, and instead automated geometric models were used to delineate these structures based on existing substructure contours (described in Sect. 2.2).

The CT imaging used in this study was acquired supine, however, arm position was variable (both arms raised, single arm raised, neither arm raised) to match the treatment position. Intravenous contrast agent was administered for a subset of lung cancer patients as per clinical protocols. For some lung patients the imaging was acquired as a 4DCT and the average intensity projection, which was used locally for radiotherapy dose calculations, was selected as the relevant image for manual contouring and automatic segmentation. Imaging for breast cancer patients was acquired as either a free breathing or deep inspiration breath hold CT. All CT images were reconstructed in axial planes with in-plane image resolution between $0.9766 \times 0.9766$ and $1.172 \times 1.172$ mm$^2$, and slice thickness between 2.0 and 2.5 mm.

### Automatic segmentation

**Fig. 2:**
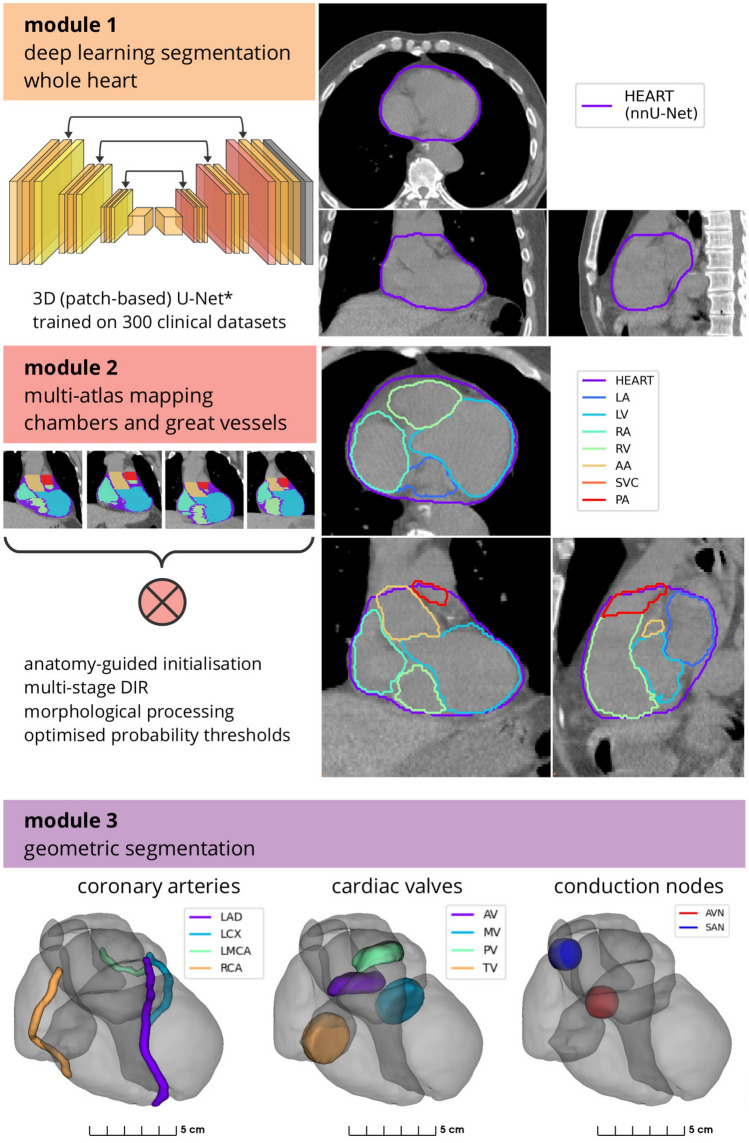
Outline of the proposed hybrid segmentation approach. The automatic cardiac substructure segmentation method comprises three modules that are used sequentially to fit a detailed model of the heart to individual patient CT imaging. First, a U-Net-based deep learning model delineates the whole heart. Second, the whole heart volume is used to guide a novel multi-atlas mapping process used to delineate the four cardiac chambers (LA, LV, RA, RV) and the bases of three cardiac vessels (AA, SVC, PA). Third, geometric modelling is used to define the coronary arteries (LAD, LCX, LMCA, RCA), heart valves (AV, MV, PV, TV), and conduction nodes (AVN, SAN). Acronyms: H - (whole) heart, LV - left ventricle, RV - right ventricle, LA - left atrium, RA - right atrium, AA - ascending aorta, PA - pulmonary artery, SVC - superior vena cava, AV - aortic valve, PV - pulmonic valve, MV - mitral valve, TV - tricuspid valve, LAD - left anterior descending coronary artery, LCX - left circumflex artery, RCA - right coronary artery, LMCA - left main coronary artery, AVN - atrioventricular node, SAN - sinoatrial node. *The nnU-Net framework [26] was used in this study

A novel hierarchical framework was designed that consisted of three distinct sequential stages, or modules: (1) DL WH segmentation, (2) WH-guided multi-atlas mapping of cardiac chambers and great vessels, and (3) geometric models of heart valves, coronary arteries, and conduction nodes. This fully-automated process is illustrated in Fig. 2.

Automatic WH segmentation was used to initialise and guide intracardiac structure segmentation. The 300 CT scans and WH contours were used to train a DL segmentation model using the state-of-the-art nnU-Net framework [15, 26, 27]. Four model types were evaluated: 2D (inference made on axial slices), 3D (inference made on 3D patches) low-resolution and full-resolution, and an ensemble model that uses weighted segmentations from each of the three previous models. Each model was trained using 5-fold cross-validation, with 1000 epochs for each fold and with the final models (for each of the four types) defined using an ensemble. Both the training and inference were executed using a NVIDIA Quadro RTX 8000 (48 GB) GPU, with a 16-core 2.2 GHz CPU and 128 GB RAM. The configuration of training parameters were set to the default settings provided by nnU-Net [26].

**Fig. 3:**
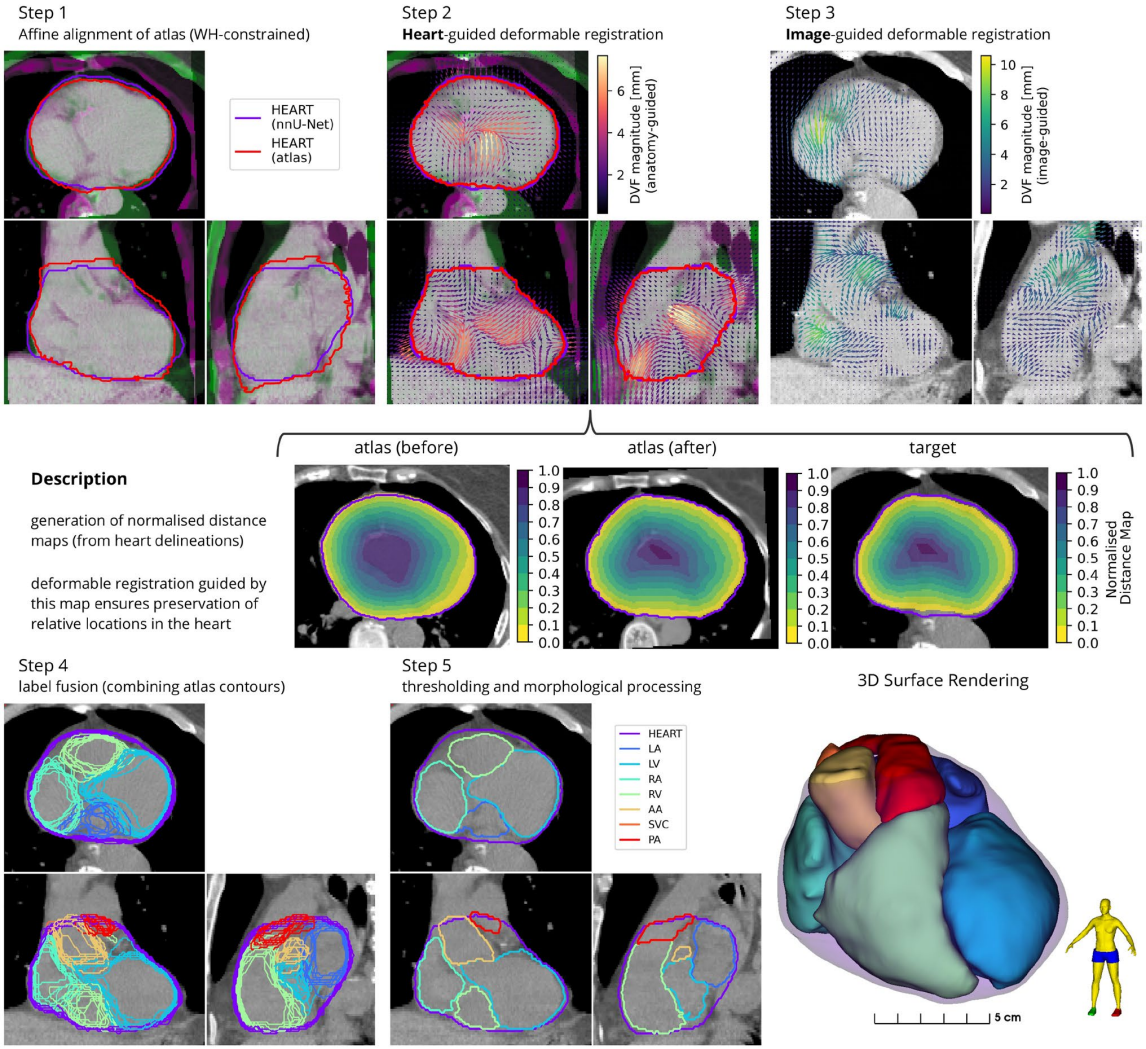
The multi-atlas mapping stage in the proposed cardiac segmentation framework was designed to delineate the heart chambers and great vessels. Three registration steps are used to co-register a set of ten atlases to the target image (top row). This included a novel WH-guided deformable registration process with distance-preserving regularisation (middle row). The atlas contours are combined using label fusion (bottom left), and then processed to produce the final contours (bottom centre and right). Acronyms: WH - whole heart, LV - left ventricle, RV - right ventricle, LA - left atrium, RA - right atrium, AA - ascending aorta, PA - pulmonary artery, SVC - superior vena cava

The second module was used to delineate seven larger cardiac substructures: the four chambers (LA, LV, RA, RV) and bases of the three great vessels (AA, PA, SVC). Atlas images and corresponding substructure contours were mapped to each target image using the automatically segmented WH as a reference. This atlas mapping used a registration framework consisting of affine alignment of atlas and target heart volumes, deformable structure-guided registration, and deformable image registration (DIR) (see Fig. 3). Details of the multi-atlas mapping method used to delineate the cardiac chambers and great vessels are provided below.

1. **Affine registration.** The atlas WH contour was registered to the DL segmentation using an affine transform. This structure-guided registration process aims to register the normalised distance maps of the target and atlas WH contours, which focuses the registration to the cardiac volume of interest and is more robust than image-based registration for this application, where differences in Hounsfield units are expected. The affine registration process accounts for gross differences in anatomy between the atlas and target patients, and initialises the next step. Registration was performed using a multi-resolution regime with 3 levels of downsampling (factors of 16,8,4), and fixed sampling rate (0.75, grid sampling), maximum number of iterations (50 per resolution level), using the mean squared difference in intensity and gradient descent line search optimisation.
2. **WH-guided deformable transformation.** A novel WH-guided registration algorithm with distance-preserving regularisation was implemented, as developed by Finnegan et al. (2022) [28]. This process also uses normalised distance maps to optimise a deformable transformation from the registered atlas WH delineation from step 1 to the automatic segmentation on the target image. Registration was performed in three resolution levels with fixed isotropic voxel sizes (16 mm, 8 mm, 3 mm) and maximum number of iterations (50 per level). As with standard structure-guided registration, this process results in near-perfect co-registration of the whole heart boundary, however it also guarantees the preservation of relative distances inside the heart volume, which was observed to provide a more consistent initialisation for the next step.
3. **Deformable image registration.** The last step in this module is deformable image registration, applied between each atlas and the target image. This implementation used a multi-resolution, log-domain, symmetric-forces diffeomorphic demons algorithm [29–31], restricted to the vicinity of the heart to reduce computational cost and prevent misregistration of the heart into nearby tissues (e.g. diaphragm, liver, stomach, lung tumours). Registration was performed in three resolution levels with fixed isotropic voxel sizes (6 mm, 3 mm, 1.5 mm) and maximum number of iterations (200, 150, 100).

Parameter selection was based on previous work [21], with modifications to resolution staging based on the physical size of anatomical and imaging features contributing to registration and with higher numbers of iterations possible due to improved algorithmic efficiency and more powerful hardware. In the multi-resolution schemes used for atlas registration, the resulting transformation at each resolution was used to initialise the next stage. Multiple atlases were registered to the target image and the set of atlas contours were combined using label fusion [32]. Post-processing included a connected components filter to remove any non-connected regions, morphological hole filling, and overlap correction (assigning the overlap region to the larger of the substructures). This implementation employed a probability optimisation scheme [21] to minimise the relative volume difference. In this study, the atlases used in this stage to generate automatic segmentations were mutually exclusive to the evaluation imaging to ensure the reliability of the experiments.

**Fig. 4:**
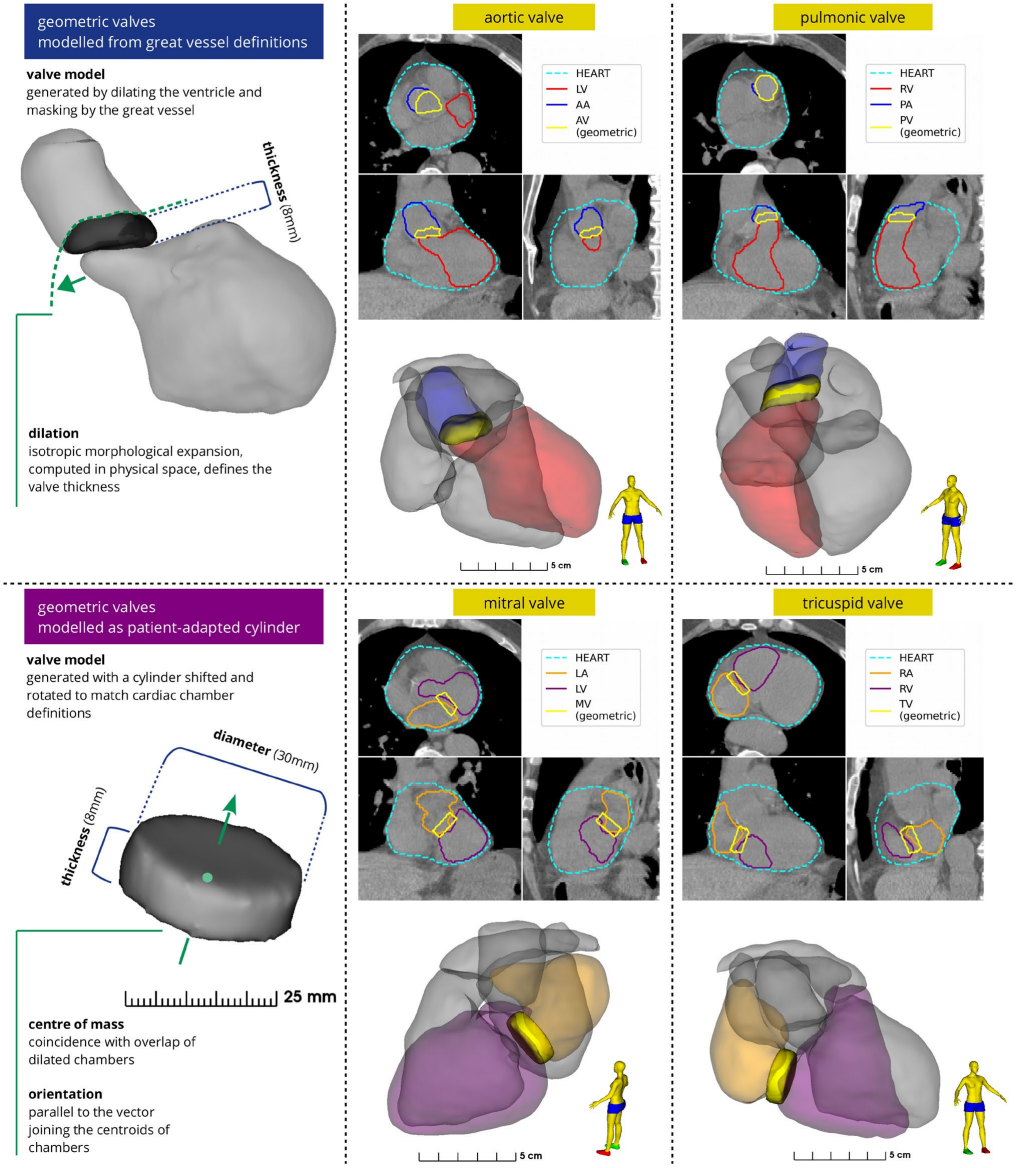
Geometric models developed in this study to define the heart valves, using delineations of other cardiac substructures. This framework includes a method to define the aortic and pulmonic valves (top), and the mitral and tricuspid valves (bottom). Acronyms: LA - left atrium, LV - left ventricle, RA - right atrium, RV - right ventricle, AV - aortic valve, PV - pulmonic valve, MV - mitral valve, TV - tricuspid valve

Heart valves and coronary arteries are difficult to visualise, especially on non-gated, non-contrast CT scans commonly used in RT planning. Thus, contouring is typically based on anatomical knowledge (i.e. where these are located relative to observable cardiac structures). This precludes consistent manual contouring of these structures, as is evident in multi-observer contouring studies [21, 24, 33] and is a primary obstacle in the development of automatic segmentation algorithms based on patient imaging. Instead, in the current work, geometric modelling was used to automatically segment the heart valves, providing anatomic consistency and uniform definitions for each patient, and a method that is independent of limitations in imaging information as it relies solely on segmentation of larger cardiac structures. Two approaches were developed to segment the heart valves, see Fig. 4. The AV and PV were defined using a dilation of the respective ventricle, masked by the corresponding great vessel. The thicknesses of the AV and PV were both set to 8 mm following recently published guidelines [33]. The MV and TV were modelled as a cylinder, which was adjusted to sit at the junction of the respective atrium and ventricle, and rotated to align with the vector directed from the centroids of these chambers (Fig. 4). For the MV and TV the diameter and thickness were set to to 30 mm and 8 mm, respectively [33]. As described in our previous work [22, 23], each coronary artery is defined as a 3D tube (diameter 4 mm [34]) constructed as a spline from the set of co-registered atlas contours.

Recently published contouring guidelines [35] provide definitions for the sinoatrial node (SAN) and atrioventricular node (AVN). To summarise: the AVN is defined as a sphere of radius 10 mm centered at the junction of the four cardiac chambers, and the SAN is defined as a sphere, also of radius 10 mm, located at the junction of the SVC and right atrium such that it does not extend beyond the whole heart volume. Using these definitions, we developed an automated geometric algorithm that delineates these structures based on the relevant cardiac substructures.

The fully-automated segmentation algorithm was written in Python 3.9 [36] and extensively uses the SimpleITK framework [37].

### Evaluation and optimisation of segmentation performance

Cardiac substructures were automatically segmented using the hybrid approach on the 30 contoured CT scans to evaluate segmentation performance. The contouring metrics used in this study were the Dice Similarity Coefficient (DSC), the mean distance to agreement (MDA), the (maximum) Hausdorff distance (HD), and volume ratio (computed as automatic/manual). The DSC is a common metric for assessing spatial overlap. The MDA quantifies overall surface-to-surface deviation between automatic and manual contours, while the HD provides an indication of the maximum deviation. The volume ratio is an important measure of contouring similarity, particularly in the context of RT where calculation of dose metrics such as mean dose can be sensitive to systematic differences in structure volumes.

To select the optimal DL model for WH segmentation, contouring metrics were computed for automatic contours generated using each of the four models. The Wilcoxon signed-rank test was used to compare performance between the models. The impact of atlas selection was tested by using three independent atlas sets. In previous studies, it has been noted that performance of automatic atlas-based cardiac segmentation methods does not increase after the number of cases in the atlas set exceeds 6–10 [38–40], and as such the number of atlases was fixed at 10. Measures of inter-observer variability were computed using pairwise comparisons between the three manual contours on the set of 20 breast cancer patient scans. The breast cancer cohort was divided into two sets of 10 cases by ranking all 20 atlases by the mean inter-observer MDA for the seven larger cardiac substructures and then selecting the 10 cases with lowest and highest variability, respectively. The 10 lung cancer patient scans comprised the third atlas set. The atlas sets are derived from the validation data, but importantly remain completely independent during the generation of automatic delineation of cardiac substructures. This was achieved using a leave-one-out approach: in situations where a test case would be included in the atlas set (e.g. automatic segmentation of a lung cancer case using the lung atlas) this test case is removed, leaving only 9 atlases.

Performance of the automatic segmentation tool was evaluated as the similarity to manual delineations. However, for smaller structures (e.g. coronary arteries and valves) where manual contouring is unreliable due to the characteristics of imaging used for RT planning, automatic segmentation performance was judged not only by similarity to manual contouring (with consideration for relatively large variability), but also by agreement with anatomical definitions (e.g. position, size, and shape of these structures) and consistency between patients.

Threshold optimisation was performed using leave-one-out analysis, and assessed by comparing the volume ratio before and after optimisation using the Wilcoxon signed-rank test. The total time taken to perform automatic segmentation was also recorded.

## Results

**Fig. 5:**
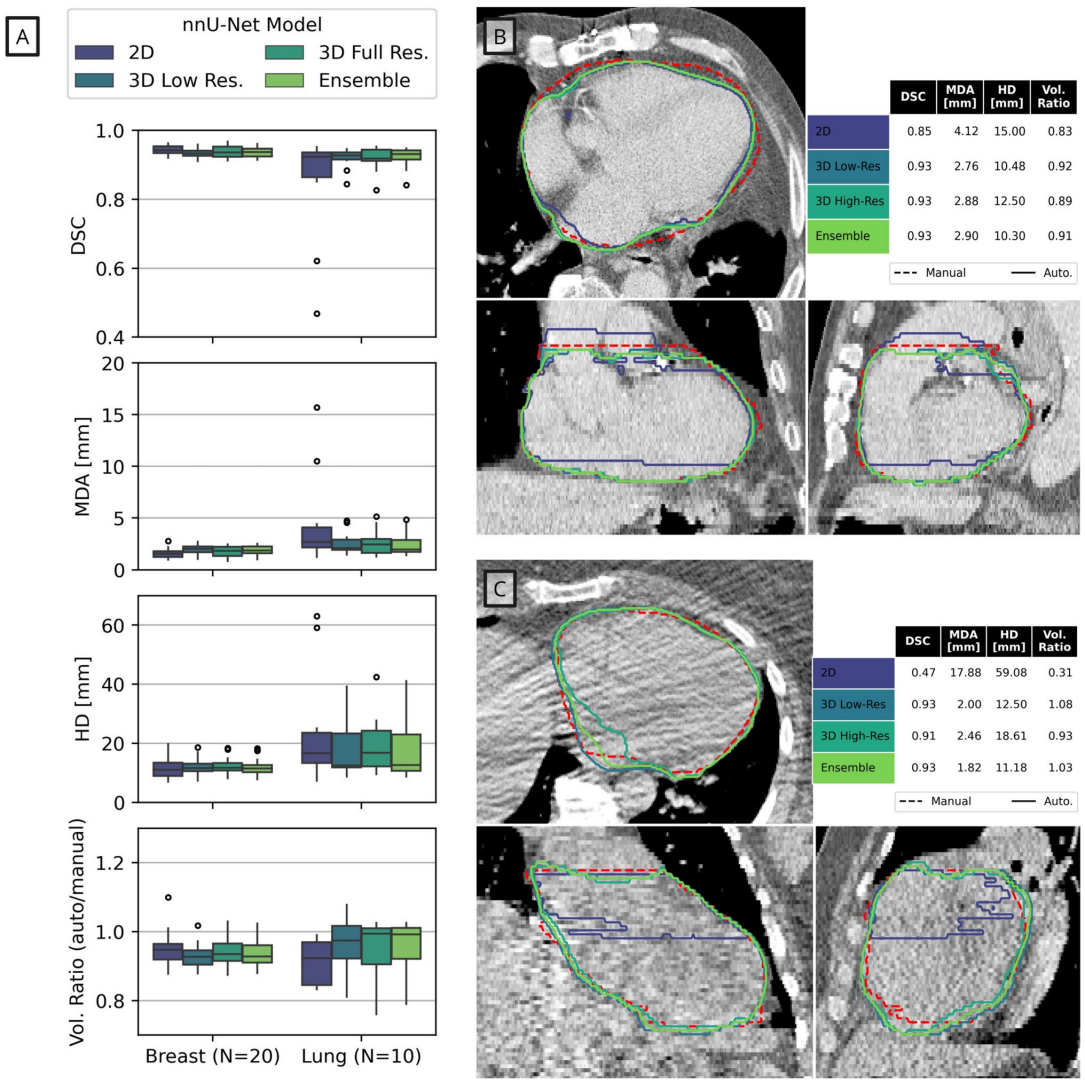
The results of the deep learning-based whole heart segmentation are summarised on the left (5A), using the Dice (DSC), mean distance to agreement (MDA), maximum Hausdorff distance (HD) and volume ratio (Vol. Ratio). Two example patients are shown on the right (5B-C), demonstrating cases where the 2D nnU-Net model failed to provide an accurate segmentation. In Figure 5A the boxes represent the first and third quartiles, the middle bars represent the medians, and the whiskers represent the range excluding any outliers which are defined as any points greater than 1.5 $\times$ the inter-quartile range above or below the first or third quartile, respectively (outliers are shown as empty circles)

Measures of contouring similarity for the four DL models for WH segmentation indicate higher performance in the breast dataset versus the lung dataset (Fig. 5A). Overall, the 2D model performed worst, with illustrative failures shown in Fig. 5B and C. There was no statistically significant difference between the remaining three models in any of the similarity metrics ($p>0.05$). Inference made with the 3D low resolution model was substantially faster and had lower variation in contouring metrics between patients. This model was selected as the optimal WH segmentation model and subsequent results are reported with the 3D low resolution model used to initialise the segmentation of cardiac substructures.

**Fig. 6:**
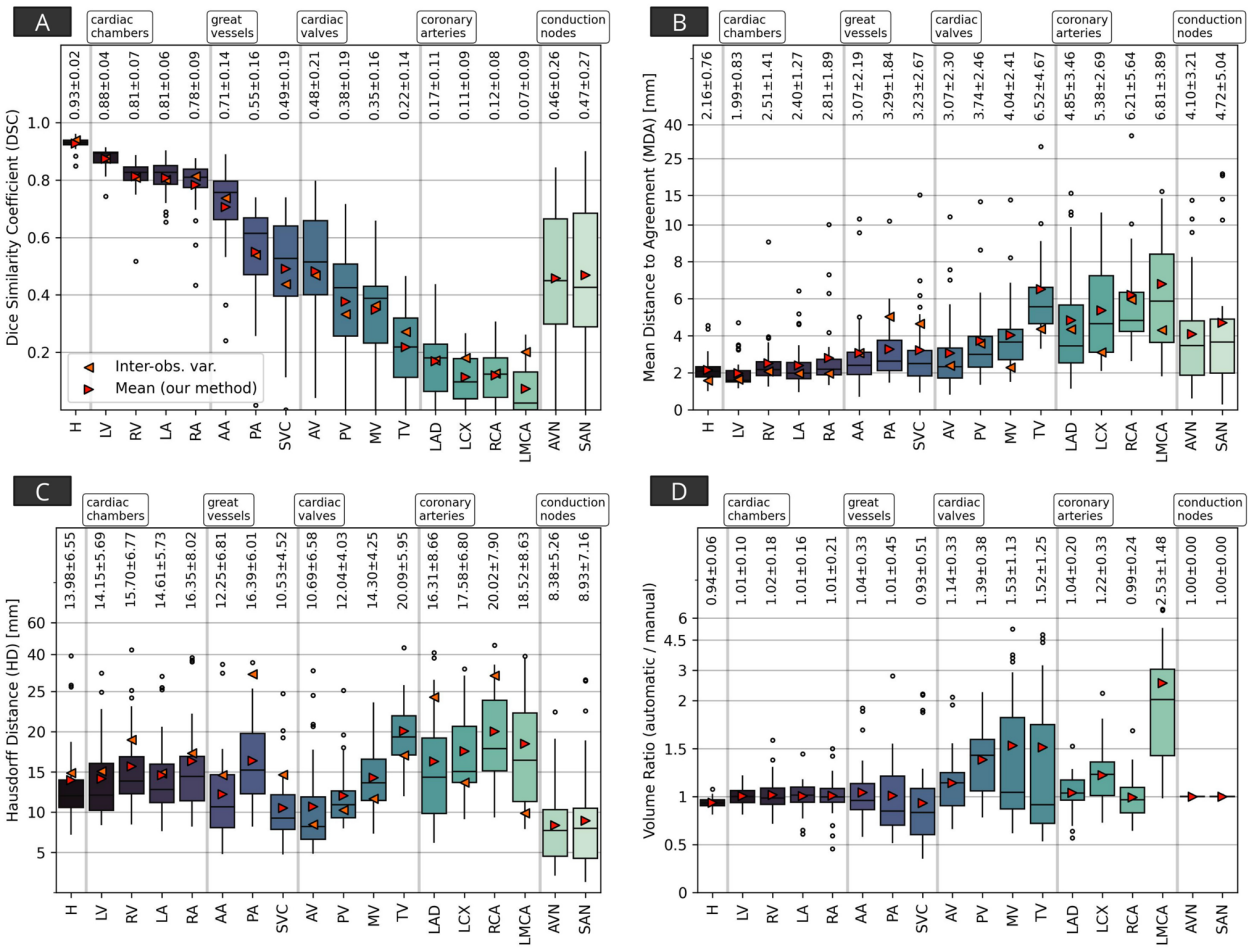
Results of comparisons between manual contours and automatic segmentations of the cardiac substructures included in this quantitative analysis, for the Dice Similarity Coefficient (DSC, 6A), the mean distance to agreement (MDA, 6B), the (maximum) Hausdorff distance (HD, 6C), and volume ratio (computed as automatic/manual, 6D). These grouped results combine both the breast and lung datasets. The measures of inter-observer contouring variability are derived from the three sets of manual contours on the breast dataset. Acronyms: H - (whole) heart, LV - left ventricle, RV - right ventricle, LA - left atrium, RA - right atrium, AA - ascending aorta, PA - pulmonary artery, SVC - superior vena cava, AV - aortic valve, PV - pulmonic valve, MV - mitral valve, TV - tricuspid valve, LAD - left anterior descending coronary artery, LCX - left circumflex artery, RCA - right coronary artery, LMCA - left main coronary artery, AVN - atrioventricular node, SAN - sinoatrial node. The boxes represent the first and third quartiles, the middle bars represent the medians, and the whiskers represent the range excluding any outliers which are defined as any points greater than 1.5 $\times$ the inter-quartile range above or below the first or third quartile, respectively (outliers are shown as empty circles). The printed text presents the mean ± standard deviation of results for each metric and substructure

Contouring metrics for all cardiac structures are presented in Fig. 6. Measures of geometric similarity indicate performance of the hybrid segmentation model is close to the level of inter-observer contouring variability, for example the median (± median absolute deviation) DSC and MDA for automatically delineated cardiac chambers was $0.83 \pm 0.07$ and $2.0 \pm 0.88$ mm compared to measured inter-observer DSC and MDA of $0.81 \pm 0.01$ and $1.9 \pm 0.09$ mm. Geometric accuracy was similar for each atlas set, however the high-variability breast atlas set generated the most accurate segmentations overall, followed by the low-variability breast atlas (see Supplementary Tables S1, S2, S3, S4). For the high-variability breast atlas set, the median DSC across the set of 30 patients ranged between 0.81–0.93 for whole heart and cardiac chambers, 0.43–0.76 for great vessels and conduction nodes, and 0.22–0.53 for heart valves. The median MDA was 2.1 mm for the WH, 1.6–2.6 mm for the cardiac chambers and great vessels, 2.3–5.6 mm for the heart valves and conduction nodes, and $3.2-5.8$ mm for the coronary arteries. The median HD ranged from 7.7 mm (AVN) to 19.7 mm (RCA), and was notably higher for coronary arteries than other types of substructures. There are outliers which suggest large discrepancies between manual and automatic delineations for a small number of cases.

**Fig. 7:**
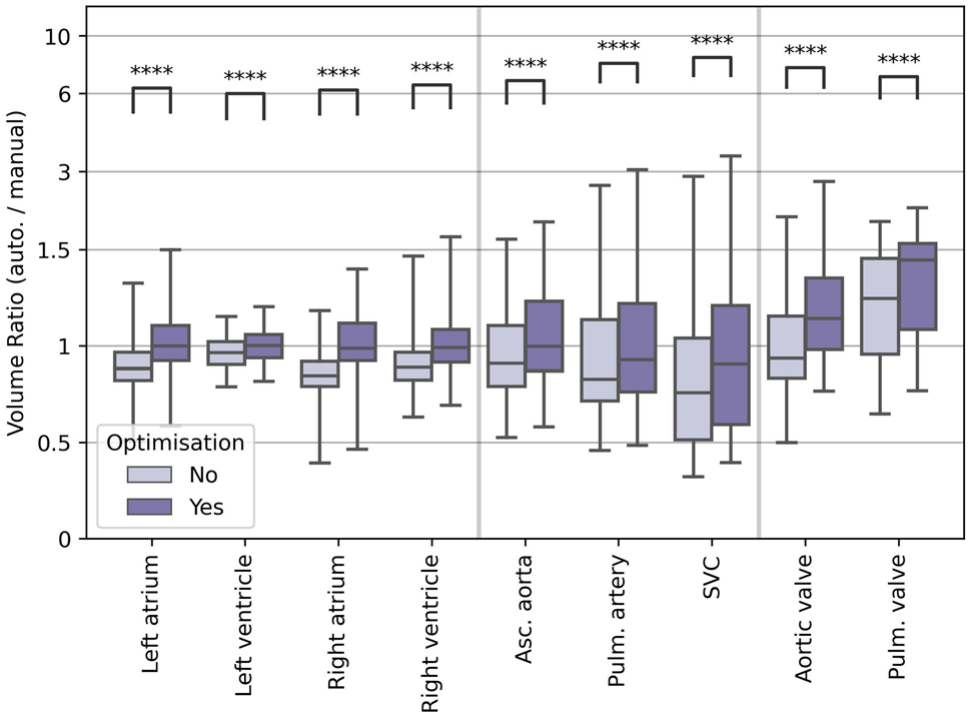
Comparison of volume ratio with and without using the probability threshold optimisation method described in Finnegan et al. [21]. The volume ratio was compared with and without using this optimisation, using the Wilcoxon signed-rank test. This optimisation process was not applied to the heart valves, but as their definition depends on structures to which optimisation was applied they are included here. The boxes represent the first and third quartiles, the middle bars represent the medians, and the whiskers represent the range excluding any outliers which are defined as any points greater than 1.5 $\times$ the inter-quartile range above or below the first or third quartile, respectively (outliers are shown as empty circles). Legend: **** = $p<10^{-4}$, Wilcoxon signed-rank test

The use of probability threshold optimisation resulted in statistically significant improvement in the volume ratio of structures it was applied to (see Fig. 7). Systematic differences remained for the structures that were not able to be directly optimised using this method (WH, valves and arteries). The median volume ratio between the automatically and manually defined WH was 0.94 (range 0.83–1.08), suggesting automatically-defined heart volumes are approximately 6% smaller than those from manual contours, and valve volumes from automatic segmentations were 26% (AV) to 53% (PV) larger than those from manual contours. Segmentation of cardiac substructures was more consistent with manual contouring in the breast cancer cohort than the lung cancer cohort (see Supplementary Fig. S1).

**Fig. 8:**
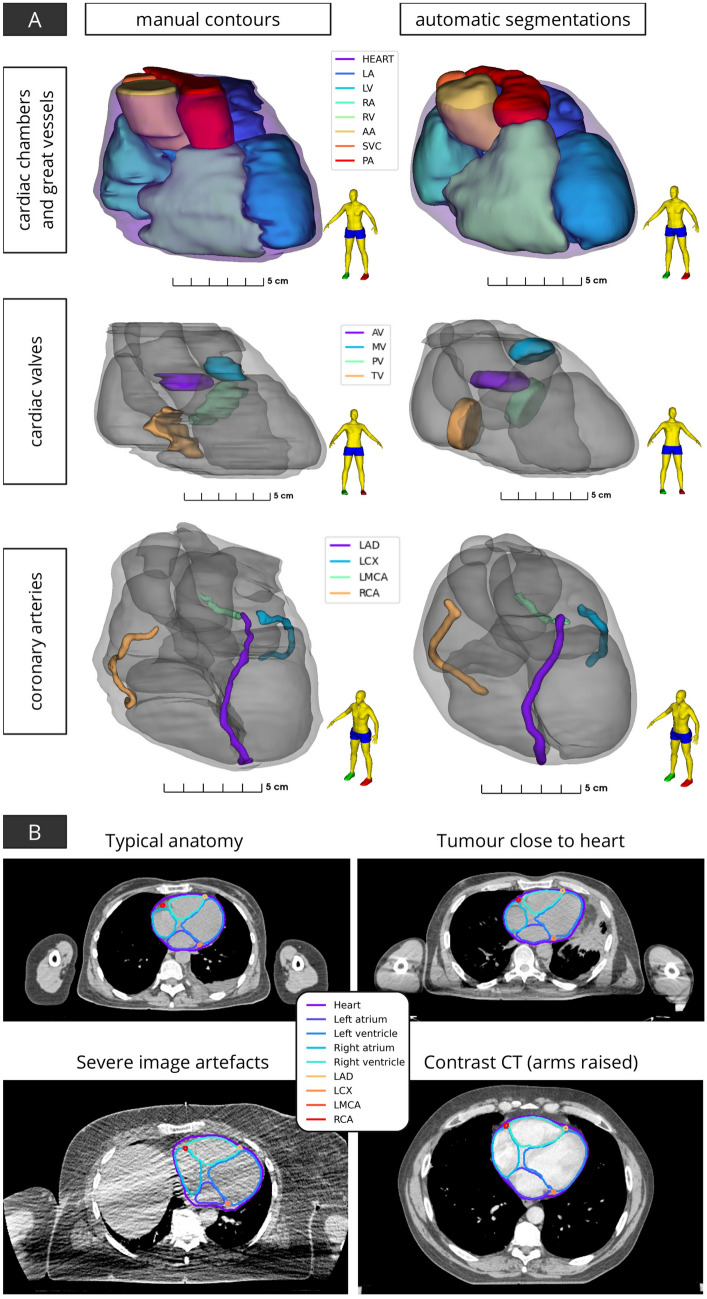
**A** Surface renderings of manual contours and automatic segmentations for a representative case (median segmentation accuracy). **B** Axial slices of radiotherapy planning CT scans shown with automatic segmentations for a number of cases with variations in imaging and patient anatomy. Acronyms: LV - left ventricle, RV - right ventricle, LA - left atrium, RA - right atrium, AA - ascending aorta, PA - pulmonary artery, SVC - superior vena cava, AV - aortic valve, PV - pulmonic valve, MV - mitral valve, TV - tricuspid valve, LAD - left anterior descending coronary artery, LCX - left circumflex artery, RCA - right coronary artery, LMCA - left main coronary artery

**Fig. 9:**
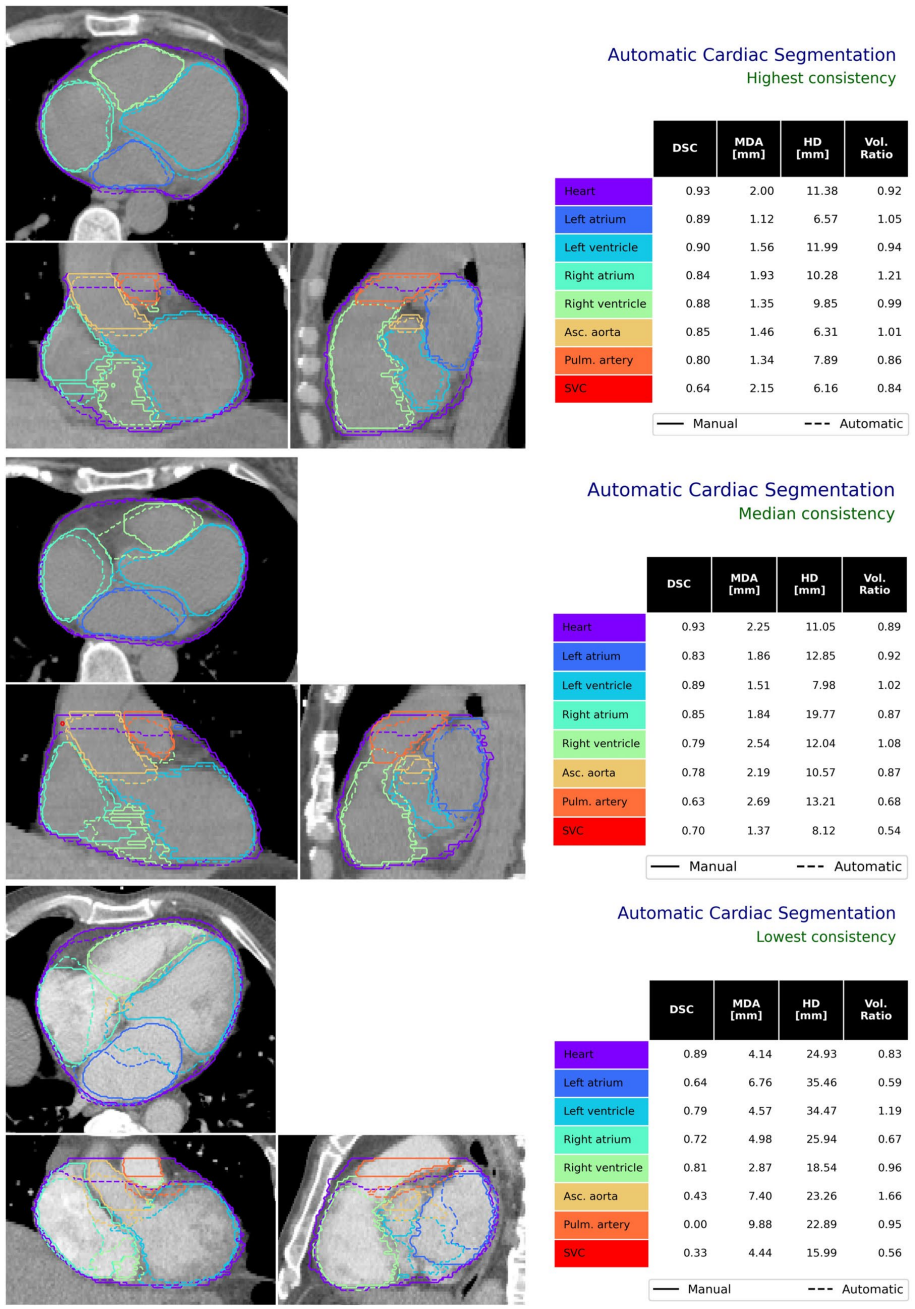
Orthogonal slices for three patient CT scans demonstrating the performance of the proposed automatic segmentation method. These are shown for patient for which the automatic delineations had the highest (top), median (middle) and lowest (bottom) consistency with manual contours. Acronyms: SVC - superior vena cava, DSC - Dice Similarity Coefficient, MDA - mean distance to agreement, HD - Hausdorff distance, Vol. - volume

Spatial smoothing of the cardiac chambers and great vessels was observed for automatic segmentations (8A). The hybrid approach was able to reliably delineate cardiac substructures despite variations in patient anatomy and imaging, as demonstrated in Fig. 8B. Manual contouring of larger cardiac structures (e.g. chambers, great vessels) is relatively consistent (low inter-observer variability). Visualisation of automatic segmentations on orthogonal slices of CT imaging is shown in Fig. 9. The time required for automatic segmentation, given as mean ± standard deviation (range), was $761\pm 109$ (563–1378)s. WH segmentation using the low resolution 3D DL model required approximately 2–5 minutes, while the full-resolution model took 2–3 $\times$ longer. The second module, multi-atlas mapping, required 5–10 min to execute and the geometric models used to delineate smaller cardiac substructures took 1–2 min. Although the DL WH segmentation model executes faster on GPU architecture (and required approximately 4 GB of memory) the entire process can also be performed on systems with only a CPU.

## Discussion

This study has designed and implemented a hybrid segmentation method, where deep learning, WH-guided multi-atlas mapping, and geometric model segmentation processes were combined to provide accurate, robust, and anatomically-consistent delineation of cardiac substructures on RT CT scans. This novel approach will facilitate analysis of large, retrospective studies to develop better risk models of radiation-related cardiotoxicities, and provides a comprehensive cardiac model comprising 18 structures.

The hybrid method leverages the strengths of different approaches to image segmentation. The WH is routinely contoured for thoracic RT patients, and the large amount of available clinical data represents a wide range of variations in patient anatomy, patient set-up, and imaging artefacts. For this reason, a DL model capable of learning from the large amount of training data is an ideal approach. To delineate larger cardiac structures (cardiac chambers and great vessels), the WH-guided multi-atlas mapping process was developed. Initialisation using the WH overcomes limitations in typical atlas-based methods which often fail when the target image is not represented in the atlas set. A disadvantage of using the WH to guide this process is that when the WH segmentation does not match the definition used in the atlas cases the performance is poor. This can be seen in the worst performing case in this study cohort, as visualised in Fig. 9), where the superior border of the DL-based WH delineation does not match the manual WH definition (which is the same as that used to produce the atlas set), and as a result the automatically segmented structures in the superior region do not match the manual definitions. A systematically smaller DL-based WH volume was observed in this study, consistent with published work [41–43], and in this case may reflect contouring variation in the training dataset.

The third module in the hybrid method contributes novel geometric modelling tools for automatic segmentation of the heart valves, conduction nodes, and coronary arteries. This overcomes challenges in manually contouring these structures due to imaging factors (e.g. lack of soft tissue contrast, motion blurring, insufficient spatial resolution), patient factors (e.g. artefacts from implants, atypical anatomy), and the oblique structure orientation relative to imaging planes. Defining conduction nodes is an important part of the development of cardiotoxicity models, as radiation-induced arrhythmias and conduction disorders are known complications following thoracic RT [44].

The evaluation of segmentation performance should be considered in the context of the imaging data used, the reliability of manual contouring, and the relevance of consistent definitions for specific structures. Multiple studies have presented quantitative data for inter-observer contouring variability for the heart and cardiac substructures [45–48]. Although direct comparison is difficult due to differences in study cohorts, image acquisition parameters, observer experience, and the specific substructures being contoured, the inter-observer variation for data used in this study [21] is in good agreement with other studies. For the CT imaging used in radiotherapy planning, a number of factors contribute to difficulty in the visualisation of smaller cardiac structures, including lack of cardiac/respiratory gating causing motion blurring, no/variable CT contrast agent use, image artefacts, atypical anatomy, and image resolution on a similar scale as the structures (e.g. slice thicknesses ranging 2–5 mm). For many patient images, this results in a high uncertainty in manual contouring, which must be considered when comparisons between automatic and manual delineations are made. Patients selected for this study were specifically chosen in order to provide challenging and variable conditions, and we expect the patient-specific uncertainties in manual contouring to be high in this cohort.

Performance of the hybrid method compares well to existing segmentation models. Recently, Jin et al. [49] demonstrated the potential of DL cardiac substructure segmentation, using a training set of non-contrast CT imaging from 60 breast cancer patients to achieve a mean DSC/MDA of 0.79/2.7 mm for cardiac chambers and 0.39/4.1 mm for smaller substructures (valves and LAD) in an independent testing dataset. This geometric accuracy is similar to our approach, with mean DSC/MDA of 0.82/2.4 mm for the chambers and 0.32/4.44 mm for the same smaller structures, while the method of Jin et al. was faster (2.1 s vs. 761 s). Using magnetic resonance imaging (MRI) alongside contrast-enhanced CT, Morris et al. developed a DL model for cardiac substructure segmentation [13] which achieved a mean DSC of 0.88 for cardiac chambers, 0.85 for great vessels and pulmonary veins, and 0.50 for the coronary arteries. Van Velzen et al (2022) [17] developed a DL model using contrast-enhanced CT scans acquired on a dual-layer CT scanner, thereby enabling the transfer of ground-truth substructure segmentations to virtual non-contrast images for applicability to RT planning scans. For larger cardiac substructures, the mean DSC/MDA was 0.76–0.88/1.7–2.7 mm, similar to our approach (0.56–0.88/1.95–3.2 mm). While these studies demonstrate the possibility of precise delineation of cardiac substructures using a DL model, both required far more than 10 labelled atlas cases used in this work to achieve similar performance. Further, the additional imaging modalities are not routinely available for the majority of radiotherapy patients.

Compared to many other studies presenting models for automatic segmentation of cardiac substructures, this work uses far less training data to achieve similar results, requiring only 10 images with manually contoured cardiac substructures compared to other approaches which used 41 [19], 127 [50], and 217 [14] cases for training. An overview of published tools for cardiac substructure segmentation can be found in a recent publication by Walls et al (Supplementary Table 11) [18]. The model proposed in this work provides automatic definitions of 18 independent structures, more than any other method currently available. A large number of cardiac substructures were included in an atlas-based automatic segmentation model developed by Maffei et al [41], however many of these are subvolumes defined geometrically, for example proximal, mid, and distal segments of the coronary arteries. Haq et al. [14] used a training set of 217 thoracic CT scans to develop a model for the heart, cardiac chambers, and great vessels (including the inferior vena cava and complete aorta extending down to the most inferior slice of the heart), and achieved better accuracy when measured with the DSC and 95th percentile of the Hausdorff distance. This method has also been validated on an independent dataset [18], with results suggesting reduced accuracy when applied to new data as well as systematic variations. A DL model developed by Garrett Fernandes et al. [50] to delineate these same cardiac substructures from a training dataset 127 CT scans was validated on an independent dataset and also achieved higher DSC values, however a substantial reduction in performance was observed on CT imaging acquired without contrast enhancement. A cascading deep learning model was recently proposed by van den Oever [19] which automatically segments the heart and chambers accurately, although this method was only tested on 6 patient CT scans. Increased volumes of training data also provide the opportunity to develop more advanced deep learning models. The recent development of a region-based fully convolutional network for cardiac substructure segmentation by Harms et al. [16], and the mutual enhancing learning-based method proposed by Momin et al. [20] deliver excellent accuracy, although it is apparent that for the coronary arteries these models do not always produce realistic segmentations.

The challenge of developing approaches for automatic segmentation of coronary arteries has led to a number of alternative approaches. Van den Bogaard et al. [51] proposed a novel geometric technique to delineate the LAD using anatomical landmarks, achieving a mean DSC of 0.15 and median average slice-wise centroid distance of 3.9 mm, similar to our work (median DSC = 0.18, median MDA = 3.2 mm). Loap et al. [52] defined a high-risk cardiac zone (HRCZ) as a surrogate volume for the LAD, which has been implemented alongside definitions of the conduction nodes in an atlas-based method [43]. While this HRCZ is an interesting and useful idea for the proposed application in breast cancer RT, it may be less effective as a surrogate for the LAD in cases where this artery is positioned in regions of steep dose gradients, such as RT for central lung tumours.

Our proposed method and study design provide a number of additional advantages. The hybrid method approach does not require any pre-processing, and can be used directly on any CT scan. Development and testing used both breast and lung cancer patients, including cases with variations in anatomy and imaging, which provides confidence in the robust performance achieved. The range of similarity metrics provides a holistic presentation of performance and offers a useful benchmark for future studies. Importantly, the open source code makes it possible for other research groups to implement, test, and validate our results.

We identified several limitations of this work. Segmentation performance was evaluated using data from a single centre, placing inherent restrictions on the generalisation to patient imaging from other institutes. A single (and different) observer contoured the 10 lung cancer patient images. Therefore, measures of inter-observer variability (computed from the 20 breast cancer patients, contoured by three observers) can only be used as a guide, although it is expected that contouring would be more variable in the lung cancer patients. The impact of blurring due to respiratory motion could contribute to the observation of lower segmentation accuracy in the lung cohort, and further investigation could simulate this effect to characterise and quantify the detriment to segmentation accuracy from this effect. Additionally, although all observers followed the same contouring guidelines, differences in how these were interpreted might have an impact on the contours. The parameters controlling the physical size of cardiac valves were identical for all patients, however future research may provide methods to individualise geometric valve definitions based on observable quantities (e.g. cardiac sizes/shapes). The conduction nodes were not manually contoured in this study and therefore results may not reflect the geometric similarity if these were defined by a manual observer. The potential impact of anatomical differences between males and females was investigated (see Supplementary Fig. S2), and although these results suggest lower geometric accuracy for images of male patients, there are only two differences across all structures and metrics that are statistically significant (DSC and MDA for the pulmonary artery). This is likely due to low numbers of patients (3 females vs. 7 males), which makes statistical assessment challenging, however it might suggest additional investigation of this effect and potential development of sex-specific segmentation models should be considered.

Future work will validate the segmentation model for use with RT data, namely to ensure consistency of dose metrics obtained from manual contours. Further research will expand our current model with additional cardiac sub-volumes, for which automatic segmentation would enable assessment of the risks of localised radiation-induced damage. These include segments of the left ventricle myocardium [53] and segments of the coronary arteries [54]. Additionally, inclusion of cardiac substructures as OARs has previously been shown to improve cardiac sparing [55], and there are plans to incorporate our automatic segmentation tool with existing RT planning software for this purpose. Finally, while improved cardiac risk models are an important step to further understanding and mitigating negative side-effects of radiotherapy, these should be considered alongside methods to limit heart dose [56, 57], and therefore additional research on the emerging radiotherapy and planning techniques with consideration of cardiac substructures is warranted.

## Conclusion

Accurate, reliable, and anatomically consistent cardiac substructure segmentation has been achieved using a hybrid approach that combines the strengths of deep learning, WH-guided multi-atlas mapping, and geometric definitions of small structures where necessary. The open-source software developed in this project will allow for analysis of large, retrospective datasets and enable improved cardiac risk modelling for radiotherapy patients.

## Supplementary Information

Below is the link to the electronic supplementary material.

Supplementary file 1 (pdf 2694 KB)

## Data Availability

All code used to generate automatic segmentations has been made available online and released as open-source software. The nnU-Net framework is available at https://github.com/MIC-DKFZ/nnUNet. Code for modules 2 (multi-atlas mapping) and 3 (geometric definitions) of the proposed approach is provided as part of the PlatiPy package, available at https://github.com/pyplati/platipy. Both software packages are available under the permissive Apache 2.0 licence (https://www.apache.org/licenses/LICENSE-2.0).
