# Supplementary material for: Open-source, fully-automated hybrid cardiac substructure segmentation: development and optimisation
Source: Phys Eng Sci Med. 2023 Feb 13;46(1):377–93. doi: 10.1007/s13246-023-01231-w (PMC10030448; doi:10.1007/s13246-023-01231-w)
Supplement: Supplementary file 1 — (pdf 2694 KB) [file 13246_2023_1231_MOESM1_ESM.pdf]

7 Supplementary Material

8 Tabulated results

Table S1: Comparison of automatic segmentation performance using the **Dice Similarity Coefficient (DSC)** using each of the three atlas sets. Results are given at the median (range) over the 30 cases in this study cohort. Acronyms: LAD - left anterior descending coronary artery, LCX - left circumflex artery, RCA - right coronary artery, LMCA - left main coronary artery, AVN - atrioventricular node, SAN - sinoatrial node.

Structure	Breast high-variability	Breast low-variability	Lung
Heart	0.93 (0.88, 0.96)	0.93 (0.88, 0.96)	0.93 (0.88, 0.96)
Left ventricle	0.89 (0.74, 0.91)	0.89 (0.73, 0.91)	0.88 (0.72, 0.90)
Right ventricle	0.83 (0.52, 0.89)	0.82 (0.53, 0.87)	0.79 (0.57, 0.84)
Left atrium	0.83 (0.65, 0.90)	0.82 (0.62, 0.88)	0.80 (0.64, 0.87)
Right atrium	0.81 (0.57, 0.87)	0.81 (0.58, 0.87)	0.75 (0.57, 0.84)
Asc. aorta	0.76 (0.24, 0.89)	0.73 (0.33, 0.90)	0.68 (0.21, 0.82)
Pulm. artery	0.62 (0.02, 0.74)	0.57 (0.00, 0.76)	0.47 (0.00, 0.78)
SVC	0.53 (0.12, 0.74)	0.52 (0.13, 0.71)	0.26 (0.00, 0.62)
Aortic valve	0.53 (0.13, 0.79)	0.54 (0.06, 0.80)	0.51 (0.03, 0.73)
Pulm. valve	0.43 (0.00, 0.71)	0.40 (0.00, 0.67)	0.34 (0.00, 0.64)
Mitral valve	0.40 (0.00, 0.66)	0.35 (0.00, 0.62)	0.34 (0.00, 0.67)
Tricuspid valve	0.22 (0.00, 0.46)	0.16 (0.00, 0.49)	0.13 (0.00, 0.43)
LAD	0.18 (0.00, 0.44)	0.19 (0.00, 0.40)	0.07 (0.00, 0.23)
LCX	0.09 (0.00, 0.26)	0.07 (0.00, 0.39)	0.07 (0.00, 0.22)
RCA	0.13 (0.00, 0.31)	0.10 (0.00, 0.28)	0.07 (0.00, 0.25)
LMCA	0.01 (0.00, 0.26)	0.00 (0.00, 0.30)	0.02 (0.00, 0.31)
AVN	0.45 (0.00, 0.84)	0.51 (0.00, 0.84)	0.41 (0.00, 0.82)
SAN	0.43 (0.00, 0.90)	0.45 (0.00, 0.93)	0.28 (0.00, 0.79)

Table S2: Comparison of automatic segmentation performance using the **mean distance to agreement (MDA) [mm]** using each of the three atlas sets. Results are given at the median (range) over the 30 cases in this study cohort. Acronyms: LAD - left anterior descending coronary artery, LCX - left circumflex artery, RCA - right coronary artery, LMCA - left main coronary artery, AVN - atrioventricular node, SAN - sinoatrial node.

Structure	Breast high-variability	Breast low-variability	Lung
Heart	2.06 (1.05, 4.56)	2.06 (1.05, 4.56)	2.06 (1.05, 4.56)
Left ventricle	1.58 (1.19, 4.70)	1.67 (1.03, 4.90)	1.87 (1.44, 5.49)
Right ventricle	2.14 (1.30, 9.07)	2.27 (1.38, 8.56)	2.65 (1.78, 6.78)
Left atrium	1.96 (1.00, 6.43)	1.95 (1.19, 6.35)	2.24 (1.26, 5.35)
Right atrium	2.17 (1.37, 10.05)	2.30 (1.40, 9.96)	2.90 (1.84, 9.39)
Asc. aorta	2.27 (0.74, 10.84)	2.52 (0.88, 9.28)	3.31 (1.49, 12.09)
Pulm. artery	2.60 (1.51, 10.51)	2.93 (1.56, 9.78)	4.36 (1.28, 12.08)
SVC	2.32 (0.97, 6986)	2.64 (1.26, 7.43)	4.61 (1.46, 11.73)
Aortic valve	2.29 (0.85, 7.57)	2.24 (0.93, 8.81)	2.53 (1.30, 11.88)
Pulm. valve	2.93 (1.39, 13.79)	3.34 (1.31, 12.45)	3.12 (1.57, 13.63)
Mitral valve	3.65 (1.53, 14.08)	3.49 (1.51, 18.17)	3.53 (1.63, 19.98)
Tricuspid valve	5.59 (3.34, 29.51)	6.05 (3.69, 29.10)	5.92 (3.04, 26.74)
LAD	3.20 (1.18, 15.50)	3.25 (1.41, 15.85)	5.26 (2.53, 12.79)
LCX	4.81 (2.13, 11.71)	4.20 (1.86, 12.92)	6.57 (3.21, 12.27)
RCA	4.68 (2.67, 34.35)	5.08 (2.59, 34.92)	6.52 (2.88, 29.05)
LMCA	5.77 (1.84, 15.85)	5.62 (1.87, 19.38)	4.94 (1.88, 12.18)
AVN	3.30 (0.64, 14.04)	3.29 (0.64, 12.85)	3.72 (1.02, 16.84)
SAN	3.61 (0.33, 20.23)	3.43 (0.23, 25.07)	4.86 (1.14, 19.79)

Table S3: Comparison of automatic segmentation performance using the **Hausdorff distance (HD) [mm]** using each of the three atlas sets. Results are given at the median (range) over the 30 cases in this study cohort. Acronyms: LAD - left anterior descending coronary artery, LCX - left circumflex artery, RCA - right coronary artery, LMCA - left main coronary artery, AVN - atrioventricular node, SAN - sinoatrial node.

Structure	Breast high-variability	Breast low-variability	Lung
Heart	12.00 (7.29, 27.06)	12.00 (7.29, 27.06)	12.00 (7.29, 27.06)
Left ventricle	11.85 (8.45, 24.90)	11.77 (7.42, 26.88)	12.66 (8.74, 32.09)
Right ventricle	13.71 (8.52, 42.35)	14.49 (8.12, 41.50)	18.61 (11.35, 39.90)
Left atrium	12.70 (7.70, 30.29)	11.78 (7.32, 30.99)	13.73 (7.98, 29.00)
Right atrium	14.03 (8.30, 38.38)	13.63 (8.96, 38.88)	16.11 (10.64, 45.75)
Asc. aorta	10.42 (4.86, 31.52)	10.52 (4.50, 29.69)	12.96 (6.96, 37.11)
Pulm. artery	15.06 (8.27, 36.08)	15.84 (8.90, 32.70)	23.86 (8.61, 41.17)
SVC	8.95 (4.80, 20.13)	9.15 (4.96, 22.36)	12.97 (5.37, 29.09)
Aortic valve	8.12 (4.88, 24.52)	8.00 (4.80, 25.48)	9.37 (6.27, 28.80)
Pulm. valve	10.87 (8.06, 25.36)	10.82 (6.04, 27.32)	13.10 (5.28, 32.76)
Mitral valve	13.35 (7.42, 23.53)	14.05 (8.74, 29.58)	14.24 (8.16, 30.38)
Tricuspid valve	19.72 (12.40, 43.53)	19.86 (12.28, 42.50)	19.64 (13.63, 39.55)
LAD	13.85 (6.25, 40.86)	15.75 (6.91, 51.39)	18.43 (11.60, 44.14)
LCX	15.06 (9.22, 33.22)	16.59 (6.96, 37.91)	19.72 (10.32, 38.72)
RCA	16.88 (9.42, 44.89)	20.02 (9.30, 45.81)	22.26 (11.83, 41.27)
LMCA	16.32 (7.98, 39.17)	12.63 (6.61, 35.26)	13.14 (6.49, 22.65)
AVN	7.68 (2.18, 22.43)	6.75 (2.18, 21.12)	8.24 (2.43, 25.40)
SAN	7.98 (1.38, 28.99)	7.58 (0.98, 33.97)	10.33 (2.80, 28.41)

Table S4: Comparison of automatic segmentation performance using the **volume ratio (automatic / manual)** using each of the three atlas sets. Results are given at the median (range) over the 30 cases in this study cohort. Acronyms: LAD - left anterior descending coronary artery, LCX - left circumflex artery, RCA - right coronary artery, LMCA - left main coronary artery, AVN - atrioventricular node, SAN - sinoatrial node.

Structure	Breast high-variability	Breast low-variability	Lung
Heart	0.94 (0.83, 1.08)	0.94 (0.83, 1.08)	0.94 (0.83, 1.08)
Left ventricle	1.00 (0.82, 1.21)	1.00 (0.83, 1.21)	0.98 (0.77, 1.25)
Right ventricle	0.99 (0.73, 1.59)	0.99 (0.70, 1.68)	0.97 (0.71, 1.60)
Left atrium	1.02 (0.61, 1.44)	1.01 (0.61, 1.49)	1.02 (0.66, 1.48)
Right atrium	1.00 (0.46, 1.50)	0.98 (0.47, 1.42)	1.01 (0.44, 1.48)
Asc. aorta	0.97 (0.61, 1.92)	1.00 (0.58, 1.92)	0.94 (0.62, 2.27)
Pulm. artery	0.85 (0.52, 2.78)	0.90 (0.49, 2.87)	0.87 (0.47, 2.77)
SVC	0.84 (0.36, 2.19)	0.84 (0.40, 2.91)	1.05 (0.35, 2.76)
Aortic valve	1.15 (0.67, 2.09)	1.18 (0.79, 2.32)	1.11 (0.65, 2.62)
Pulm. valve	1.45 (0.79, 2.22)	1.49 (0.76, 2.23)	1.60 (0.87, 4.36)
Mitral valve	1.04 (0.63, 5.20)	1.04 (0.63, 5.18)	1.04 (0.63, 5.20)
Tricuspid valve	0.89 (0.54, 4.81)	0.89 (0.54, 4.80)	0.89 (0.54, 4.81)
LAD	1.05 (0.57, 1.52)	0.98 (0.53, 1.33)	1.12 (0.54, 1.57)
LCX	1.24 (0.73, 2.21)	1.03 (0.64, 1.73)	1.18 (0.60, 2.13)
RCA	0.96 (0.65, 1.69)	0.97 (0.61, 1.71)	1.07 (0.60, 2.15)
LMCA	2.07 (0.99, 6.76)	1.71 (0.94, 5.60)	0.98 (0.50, 2.91)
AVN	1.00 (1.00, 1.00)	1.00 (1.00, 1.00)	1.00 (1.00, 1.00)
SAN	1.00 (1.00, 1.00)	1.00 (1.00, 1.00)	1.00 (1.00, 1.00)

9 Additional figures

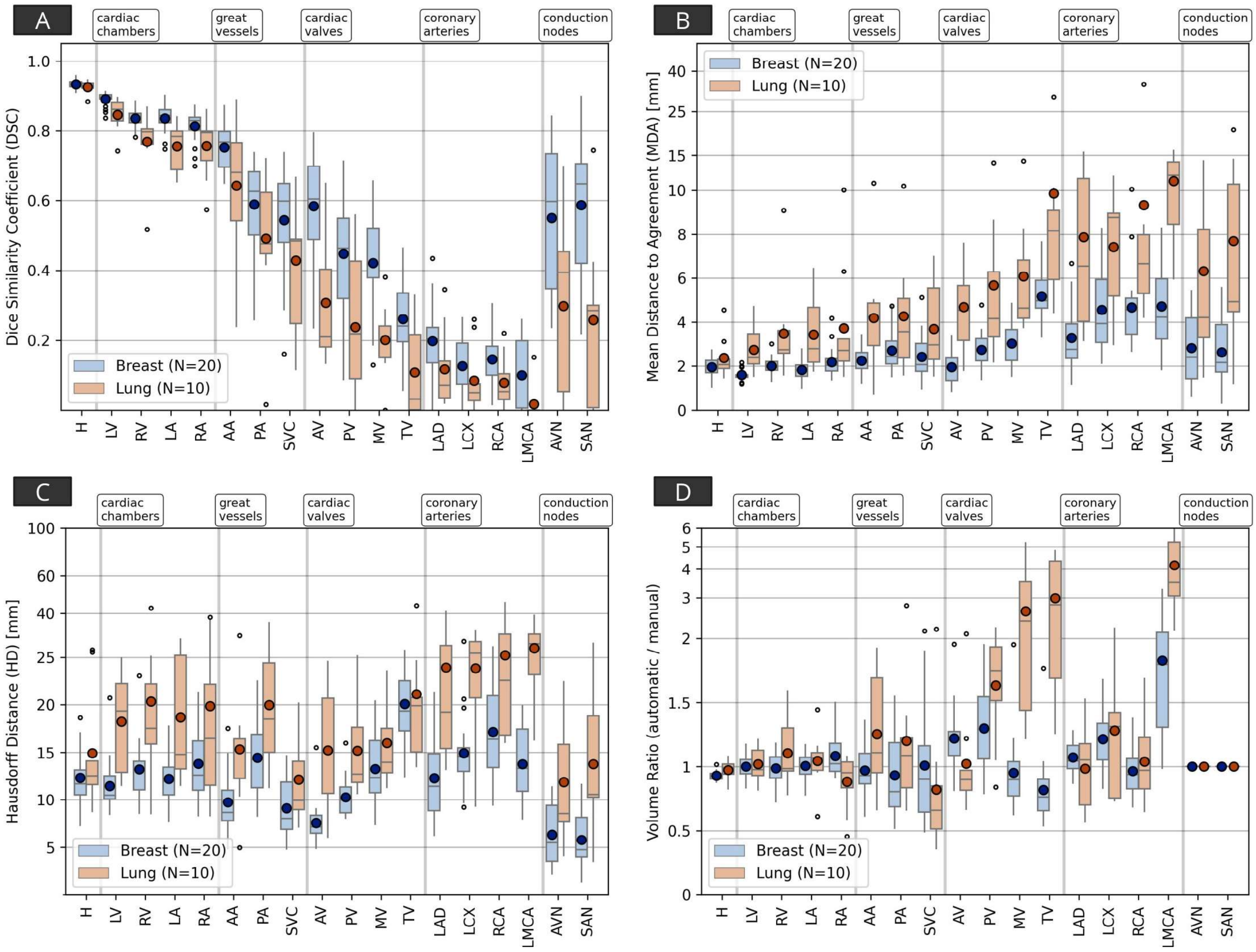

Figure S1: Results of comparisons between manual contours and automatic segmentations of the cardiac substructures included in this quantitative analysis, for the Dice Similarity Coefficient (DSC, A), the mean distance to agreement (MDA, B), the (maximum) Hausdorff distance (HD, C), and volume ratio (computed as automatic/manual, D). The solid circle in each boxplot represents the mean. These results are computed separately for the breast (N=20) and lung (N=10) datasets. Acronyms: H - (whole) heart, LV - left ventricle, RV - right ventricle, LA - left atrium, RA - right atrium, AA - ascending aorta, PA - pulmonary artery, SVC - superior vena cava, AV - aortic valve, PV - pulmonic valve, MV - mitral valve, TV - tricuspid valve, LAD - left anterior descending coronary artery, LCX - left circumflex artery, RCA - right coronary artery, LMCA - left main coronary artery, AVN - atrioventricular node, SAN - sinoatrial node.

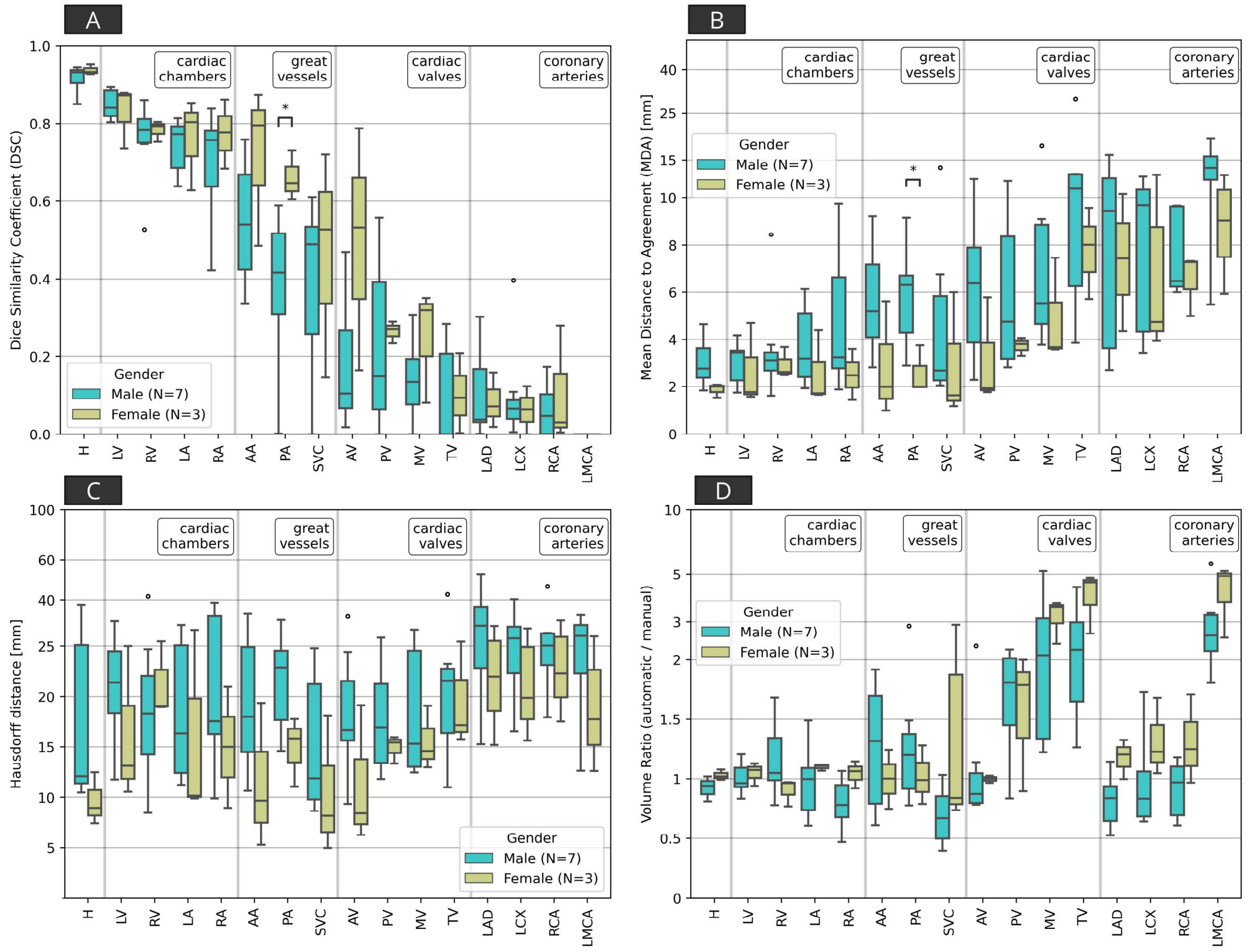

Figure S2: Results of comparisons between manual contours and automatic segmentations of the cardiac substructures for the lung cancer patient cohort, grouped by patient sex (male or female). Acronyms: H - (whole) heart, LV - left ventricle, RV - right ventricle, LA - left atrium, RA - right atrium, AA - ascending aorta, PA - pulmonary artery, SVC - superior vena cava, AV - aortic valve, PV - pulmonic valve, MV - mitral valve, TV - tricuspid valve, LAD - left anterior descending coronary artery, LCX - left circumflex artery, RCA - right coronary artery, LMCA - left main coronary artery, AVN - atrioventricular node, SAN - sinoatrial node. The boxes represent the first and third quartiles, the middle bars represent the medians, and the whiskers represent the range excluding any outliers which are defined as any points greater than 1.5 $\times$ the inter-quartile range above or below the first or third quartile, respectively (outliers are shown as empty circles). Statistically significant differences in metrics between male and female groups are shown with an asterisk (defined as Mann-Whitney U rank test p-value < 0.05).
